# Mesangial cell-derived CircRNAs in chronic glomerulonephritis: RNA sequencing and bioinformatics analysis

**DOI:** 10.1080/0886022X.2024.2371059

**Published:** 2024-07-01

**Authors:** Ji Hui Fan, Xiao Min Li

**Affiliations:** a Department of Nephrology, Huaibei People’s Hospital, Huaibei, China; b Department of Traditional Chinese Medicine, Huaibei People’s Hospital, Huaibei, China

**Keywords:** CircRNA, CeRNA, MMCs, CGN, RNA-seq, GEO

## Abstract

**Background:**

Circular RNAs (circRNAs) have been shown to play critical roles in the initiation and progression of chronic glomerulonephritis (CGN), while their role from mesangial cells in contributing to the pathogenesis of CGN is rarely understood. Our study aims to explore the potential functions of mesangial cell-derived circRNAs using RNA sequencing (RNA-seq) and bioinformatics analysis.

**Methods:**

Mouse mesangial cells (MMCs) were stimulated by lipopolysaccharide (LPS) to establish an *in vitro* model of CGN. Pro-inflammatory cytokines and cell cycle stages were detected by Enzyme-linked immunosorbent assay (ELISA) and Flow Cytometry experiment, respectively. Subsequently, differentially expressed circRNAs (DE-circRNAs) were identified by RNA-seq. GEO microarrays were used to identify differentially expressed mRNAs (DE-mRNAs) between CGN and healthy populations. Weighted co-expression network analysis (WGCNA) was utilized to explore clinically significant modules of CGN. CircRNA-associated CeRNA networks were constructed by bioinformatics analysis. The hub mRNAs from CeRNA network were identified using LASSO algorithms. Furthermore, utilizing protein–protein interaction (PPI), gene ontology (GO), pathway enrichment (KEGG), and GSEA analyses to explore the potential biological function of target genes from CeRNA network. In addition, we investigated the relationships between immune cells and hub mRNAs from CeRNA network using CIBERSORT.

**Results:**

The expression of pro-inflammatory cytokines IL-1β, IL-6, and TNF-α was drastically increased in LPS-induced MMCs. The number of cells decreased significantly in the G1 phase but increased significantly in the S/G2 phase. A total of 6 DE-mRNAs were determined by RNA-seq, including 4 up-regulated circRNAs and 2 down-regulated circRNAs. WGCNA analysis identified 1747 DE-mRNAs of the turquoise module from CGN people in the GEO database. Then, the CeRNA networks, including 6 circRNAs, 38 miRNAs, and 80 mRNAs, were successfully constructed. The results of GO and KEGG analyses revealed that the target mRNAs were mainly enriched in immune, infection, and inflammation-related pathways. Furthermore, three hub mRNAs (BOC, MLST8, and HMGCS2) from the CeRNA network were screened using LASSO algorithms. GSEA analysis revealed that hub mRNAs were implicated in a great deal of immune system responses and inflammatory pathways, including IL-5 production, MAPK signaling pathway, and JAK-STAT signaling pathway. Moreover, according to an evaluation of immune infiltration, hub mRNAs have statistical correlations with neutrophils, plasma cells, monocytes, and follicular helper T cells.

**Conclusions:**

Our findings provide fundamental and novel insights for further investigations into the role of mesangial cell-derived circRNAs in CGN pathogenesis.

## 1. Introduction

Chronic glomerulonephritis (CGN), the most prevalent kidney disease, afflicts a substantial proportion of the global chronic kidney disease population [1,2]. CGN is an immune disease characterized by the infiltration of inflammatory cells and the accumulation of extracellular matrix (ECM) [3]. Three different types of cells (mesangial cells, podocytes, and endothelial cells) make up the glomerulus [4]. The outcome of CGN is mainly determined by resident glomerular cells, especially mesangial cells, which play a critical role in glomerular injury [5,6]. Therefore, inflammation and proliferation of mesangial cells are important pathological features of many human kidney diseases, including CGN and diabetic nephropathy. In-depth exploration of the pathological features of mesangial cells is crucial for studying the occurrence and development of CGN.

Leonardo Salmena first proposed the CeRNA theory in 2011, and it is widely accepted among scientists working on non-coding RNA. The CeRNA theory allows circRNAs to function as miRNA sponges, competing with their response elements for binding and inhibiting their expression and function [7,8]. For example, CircMTO1 acts as a sponge for MIR-9, inhibiting the occurrence and development of liver carcinoma [9]. However, the ceRNA regulatory network associated with circRNAs has not been well understood in CGN.

**Figure 1.**
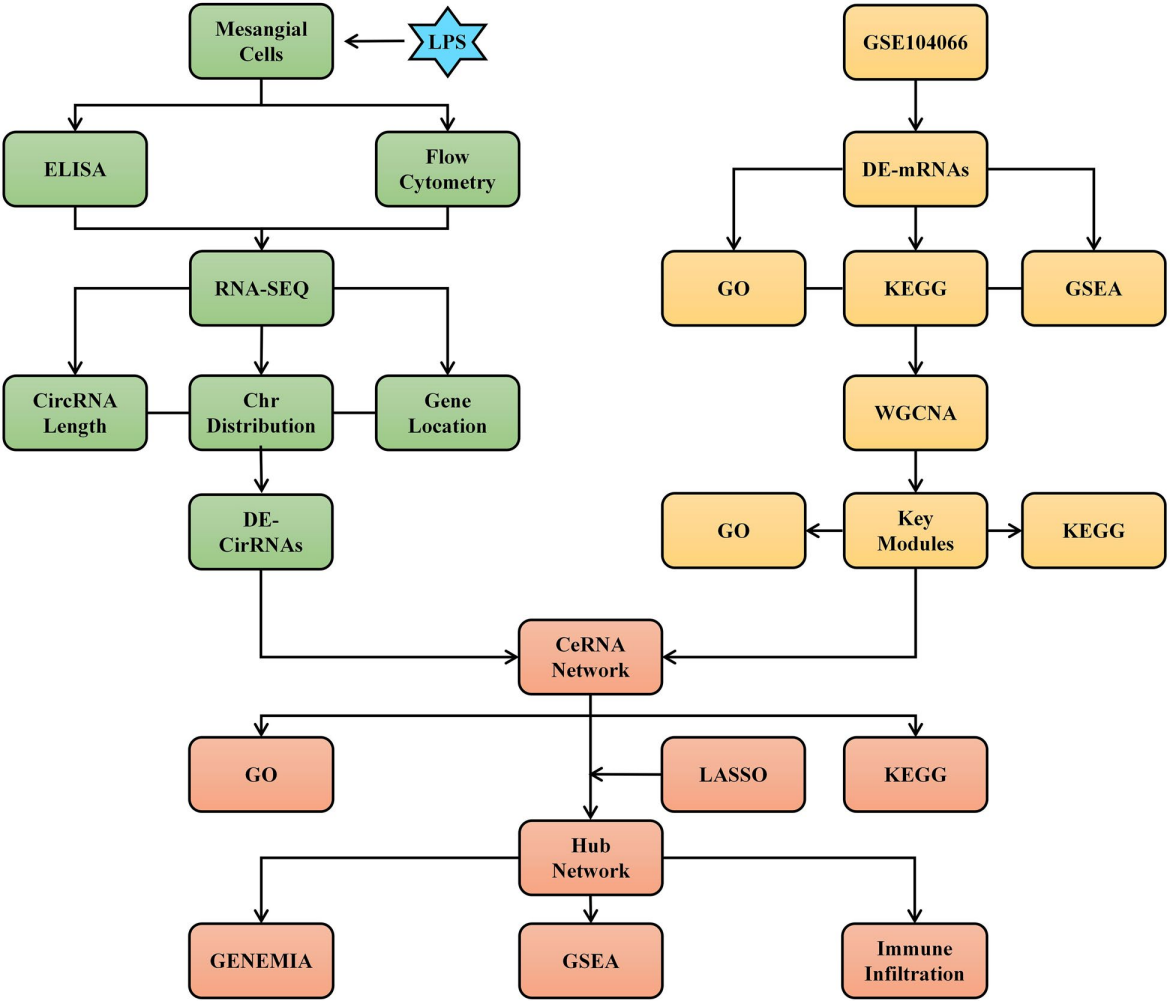
The study flowchart for mesangial cell-derived CircRNAs in CGN using RNA sequencing and bioinformatics analysis.

In our study, we evaluated the circRNA expression profile and identified DE-circRNAs in MMCs following LPS treatment. Based on the GEO database and WGCNA analysis, we identified DE-mRNAs in CGN. We further constructed the CircRNA-mediated CeRNA network and identified the network’s hub mRNAs using the LASSO methods. GO and KEGG showed that CeRNA networks were mainly involved in infection, inflammation, and autoimmunity. Moreover, GSEA analysis revealed that hub mRNAs from CeRNA networks were substantially implicated in diverse immunological and inflammatory responses. In addition, we utilized CIBERSORT to determine the association between immunity and hub mRNAs. We hope our work will provide a new perspective to further investigate the potential roles of the novel circRNAs from mesangial cells in the pathogenic mechanism of CGN. The study flowchart is depicted in Figure 1.

## 2. Materials and methods

### 2.1. Cell line and cell culture

SV40-MES-13 mouse mesangial cells (MMCs) were procured from BNCC Biological Technology (Beijing, China) and cultured in DMEM medium (Solarbio) enriched with 10% fetal bovine serum (FBS; BI) and penicillin/streptomycin at 37 °C with 5% CO_2_ in a humidified atmosphere. Subsequently, the MMCs were categorized into two groups. In the LPS group (*n* = 3), LPS (3 µg/mL) (Solarbio) was added to the MMCs. Whereas in the control group (*n* = 3), no addition was made to the MMCs. After a 24-h incubation period, the MMCs were harvested and used for the subsequent experiments.

### 2.2. Enzyme-linked immunosorbent assay (ELISA)

According to manufacturer protocols, we used specific ELISA kits obtained from Multisciences Biotech, Ltd. to detect the levels of inflammatory factors interleukin‑1β (IL‑1β) (A101B10151 and EK101B-24), IL-6 (interleukin-6) (A10610321 and EK106/2-24), and tumor necrosis factor-α (TNF-α) (A18210342 and EK182-24) after the cell-culture supernatants were collected. Absorbance at 450 nm was then measured using a Multiskan Spectrum Microplate Reader (Thermo Fisher Scientific, Inc., Waltham, MA).

### 2.3. Flow cytometry experiment

The cells in each group were rinsed using sterile phosphate-buffered saline (PBS) before being treated with 1 mL of trypsin for 2 min, followed by centrifugation at 2000 rpm for 5 min. The resulting supernatant was then collected and fixed at −20 °C in pre-chilled absolute ethanol for 1 h. After being washed twice with cold PBS, 20 µL RNase was added to the fixed cells. The cells were incubated in a water bath at 37 °C for 30 min, and then 400 μL of PI dyes (BB20071 and BB-4104) were added to collect and stain the cells at 4 °C for 40 min. Subsequently, flow cytometry (BECKMAN, Brea, CA) was used for cell cycle analysis, and the percentage of MMCs at all stages was assessed and visualized using Flow JO version 7.6 (Ashland, Oregon, USA).

### 2.4. CircRNA sequencing analysis

We used FastQC software version 0.10.1 (Cambridge, Cambridgeshire, UK) to evaluate the quality of paired-end sequencing reads generated from high-throughput RNA-Seq performed by Genesky Biotechnologies Inc. (Shanghai, China). RNA fragments were fragmented using Bioruptorpico and sonicated in RNase-free water, and their quality was assessed by denaturing gel electrophoresis. The quantity of RNA was measured using the NanoDrop 2000 Spectrophotometer (Thermo Fisher Scientific). Illumina TruSeq RNA sample preparation kits (Illumina, San Diego, CA) were constructed by using purified RNA fragments. Quality control was performed in the library and quantification was performed using the gilent 2100 Bioassay System. The sequence was performed using an Illumina HiSeq 2500 instrument with a length of 150 bp pair-end. CIRCexplorer version 2 software (Tsinghua University, Beijing, China) was used to predict the start and end positions of circRNA and the gene annotation of the source.

### 2.5. GEO public database analysis

The NCBI GEO GSE104066 datasets provided data with clinical details on CGN and healthy kidney samples. The 64 CGN kidney tissues and 6 normal tissues in the GSE104066 datasets were based on the Affymetrix Human Gene 2.1 ST Array of the GPL19983 platform. The limma R tool was then used in differential analysis to find the genes that differed between the CGN group and the control group [10]. | Fold-change (FC) | > 1.5 and *p* value < 0.05 were the statistical thresholds for screening RNA expression.

### 2.6. Weight gene correlation network analysis (WGCNA)

Using gene expression profiles, we determined the mean absolute deviation (MAD) for each gene and excluded the bottom 50% of DEGs with the lowest MAD. Additionally, the R package WGCNA was utilized to eliminate outlier DEGs and probes to build a scale-free co-expression network. Specifically, Pearson’s correlation matrices and average linkage methods were used for all gene pairs. The parameter β served as a soft-threshold to enhance strong correlations and penalize weak ones. Choosing a power of 6, the adjacency was converted into a topological overlap matrix (TOM), which measured network connectivity of a gene as the sum of its adjacency with all other genes, and calculated the corresponding dissimilarity (1-TOM). Genes with similar expression profiles were classified into modules through average linkage hierarchical clustering based on the TOM dissimilarity measure, with a minimum module size of 10 genes. For further module analysis, the dissimilarity of module eigengenes was calculated, a cut line for the module dendrogram was chosen, and some modules were merged.

### 2.7. Construction of the CircRNA-mediated CeRNA networks

To construct the circRNA-miRNA interaction network, we employed the CeRNA theory and utilized CircAtlas version 2.0 software (http://circatlas.biols.ac.cn/). We predicted target mRNAs for the identified miRNAs using TargetScan (https://www.targetscan.org/). Only target genes found in both the predicted mRNAs of TargetScan and the DE-mRNAs of GEO database were selected to construct the interaction network. The network was visualized using Cytoscape version 3.8.1 software (San Diego, California, USA).

### 2.8. Identification of hub mRNAs from the CeRNA network based on machine learning algorithm

Then, the hub mRNAs from CeRNA network were found using Cox regression with the Least Absolute Shrinkage and Selection Operator (LASSO). Based on the 3-fold cross-validation method, we calculated the penalty parameter, selected the best value corresponding to the lowest cross-validation error, and listed the gene names matching that value utilizing the ‘glmnet’ software package.

### 2.9. GO and KEGG analysis

The ClusterProfiler R package version 3.6.0 (University of Auckland, Auckland, New Zealand) was employed for conducting GO and KEGG functional enrichment analyses [11]. A p-value was computed, and a filtering threshold of *p* < 0.05 was selected. The GeneMANIA database (http://genemania.org/) is a website for building PPI networks. Using GeneMANIA, we identified PPI networks of hub biomarkers in this study.

### 2.10. Gene set enrichment analysis (GSEA)

To investigate the potential roles of selected biomarkers in CGN, GSEA analysis was performed to explore GO items and KEGG pathways [12]. Samples were divided into low-expression groups (50%) and high-expression groups (50%) based on the expression levels of the biomarkers. Reference gene sets included the Molecular Signatures Database datasets c2.cp.kegg.version 7.4.symbols.gmt and c5.hpo.version 7.4.symbols.gmt (Cambridge, Massachusetts, USA). For GSEA analysis with default settings, *p* < 0.05 was regarded as statistically significant.

### 2.11. Evaluation of immune cell infiltration and correlation analysis between hub mRNAs from the CeRNA network and infiltrating immune cells

In order to estimate the frequency of immunological invasion, the 1000-permutation deconvolution method CIBERSORT converts the expression matrix into different immune cell types [13]. Then, generate a histogram to illustrate the different cell components. A correlation heatmap of different cell components was created to show associations between different subtypes. A box plot was also used to show the differential analysis between immune cells from CGN and healthy tissue. A correlation analysis of the Spearman’s rank was utilized to examine and illustrate relationships between the detected biomarkers and the quantity of invading immune cells.

## 3. Results

### 3.1. Establishment of an *in vitro* model of LPS-induced inflammation in mesangial cells

**Figure 2.**
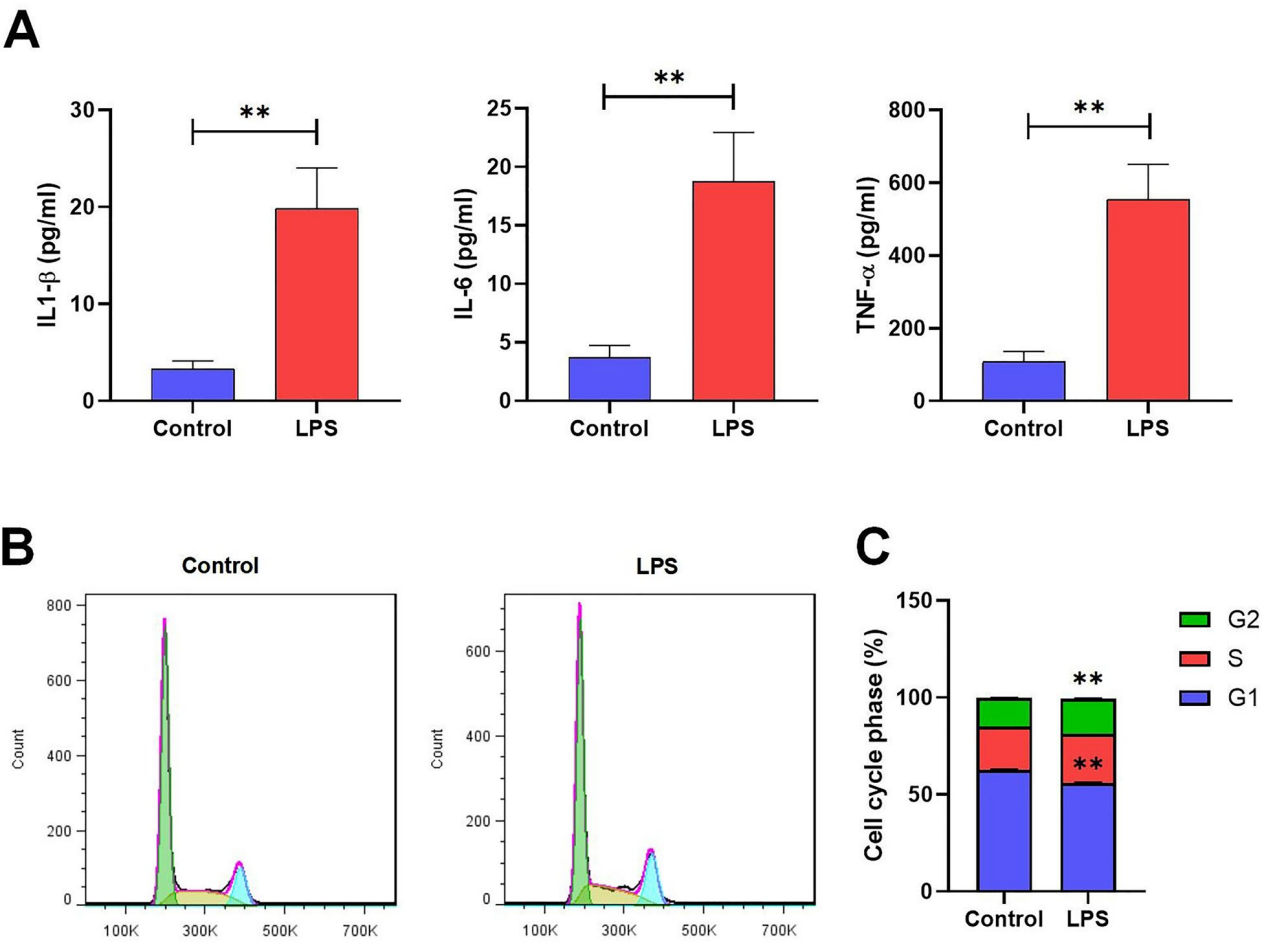
The level of inflammation and proliferation in MMCs. (A) ELISA assay measuring the concentration of IL-1β, IL-6, and TNF-α in the supernatant of MMC cells (*p < 0.05, **p < 0.01). (B,C) Flow cytometry identifying the phase of the cell cycles (*p < 0.05, **p < 0.01).

To investigate the possible biological role of circRNAs in the pathogenesis of CGN, we used LPS-induced MMCs to mimic the pathological abnormalities in this study. As expected, the pro-inflammatory cytokines IL‑1β, IL-6, and TNF-α were observed to increase significantly in the LPS group compared to those in the control group (Figure 2(A)) (Sup. File 1). Afterward, a flow cytometry analysis was conducted to assess differences in the cell cycle of MMCs. Results showed that MMCs in the LPS group had a significantly lower number of G1 cells compared to those in the control group. Moreover, there was a substantial increase in the number of cells in the S/G2 phases compared to the control group (Figure 2(B,C)) (Sup. File 2).

### 3.2. Global CircRNA distribution analysis and identification of differentially expressed CircRNAs (DE-circRNAs)

**Figure 3.**
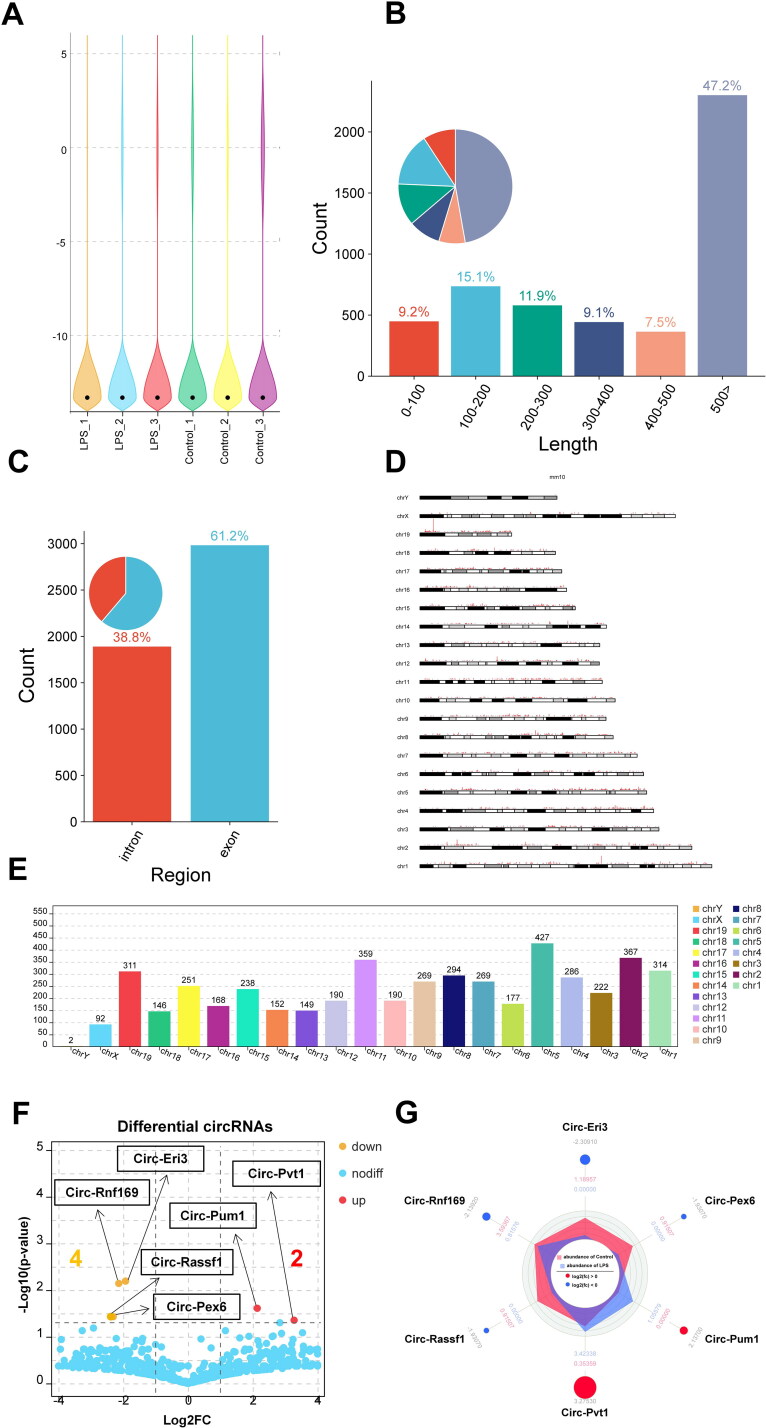
A General overview of the CircRNA distribution map in MMCs. (A) Violin plot showing the relative abundance of circRNAs in six sample cells. (B) The length distribution range of circRNAs in MMC cells. (C) Percentage pie charts of circRNAs in MMC cells. (D,E) Distribution sites of circRNAs in 22 chromosomes, as well as the number of circRNAs in each chromosome of MMC cells. (F) Volcano plots showing significantly differential circRNAs (|FC| > 1.5 and *p* < 0.05) between LPS-stimulated MMCs and normal MMCs. (G) The radar map showing the expression abundance and enrichment multiples of significantly differential circRNAs.

In this study, RNA-seq assays were conducted on normal MMCs (*n* = 3) and LPS-induced MMCs (*n* = 3). In total, 4768 circRNAs were identified across all six samples, and detailed information about each circRNA can be found in Sup. Files 3 and 4. The expression levels of circRNAs were depicted in a violin plot, as shown in Figure 3(A). We next calculate the length distribution range of circRNAs. As shown in Figure 3(B), the length of circRNAs that accounts for the maximum percentage (47.2%) is 500 bp or more, while the length that accounts for the minimum percentage (7.5%) is 400–500 bp. In addition, the length of the circRNA range at 0–400 bp accounts for 9.2%, 15.1%, 11.9%, and 9.1%, respectively.

To explore the distribution of circRNAs across transcripts, we examined the circRNA profiles within the entire transcriptome. As shown in Figure 3(C), the vast majority of circRNAs were found to be exonic (61.2%) or intronic (38.8%). Further analyses were conducted to examine the distribution of circRNAs across all chromosomes (chr). Results indicated that circRNAs were distributed across all chr to varying extents (Figure 3(D)). The top 5 chr with the highest number of circRNAs were chr5 (427), chr2 (367), chr11 (359), chr1 (314), and chr19 (311) (Figure 3(E)).

For the differential expression of circRNAs detected by RNA-seq (Sup. File 5), the volcano plot visually displayed 6 DE-circRNAs (*p* < 0.05 and |fold change| > 2), of which 2 were up-regulated and 4 were down-regulated (Figure 3(F)) (Table 1). Similarly, the corresponding expression abundance and enrichment multiples of 6 DE-circRNAs between normal MMC cells and LPS-induced MMC cells were also displayed *via* radar map in Figure 3(G).

**Table 1.** List of the differentially up-regulated and down-regulated circRNAs.

Gene ID	chr	chromStart	chromEnd	Location	*p* Value	Log2FC	Type
*Circ-Pvt1*	chr15	62107148	62107465	Exon	0.044200327	3.2753	Up
*Circ-Pum1*	chr4	1307279988	130730548	Exon	0.024513764	2.137	Up
*Circ-Pex6*	chr17	46723736	46723899	Intron	0.006415846	−1.9307	Down
*Circ-Rassf1*	chr9	107557558	107557747	Intron	0.006415846	−1.9307	Down
*Circ-Rnf169*	chr7	99935162	99955540	Exon	0.007201826	−2.1392	Down
*Circ-Eri3*	chr4	117582600	117593162	Exon	0.037139688	−2.3091	Down

### 3.3. Identification of differentially expressed mRNAs (DE-mRNAs) of CGN from the GEO database

**Figure 4.**
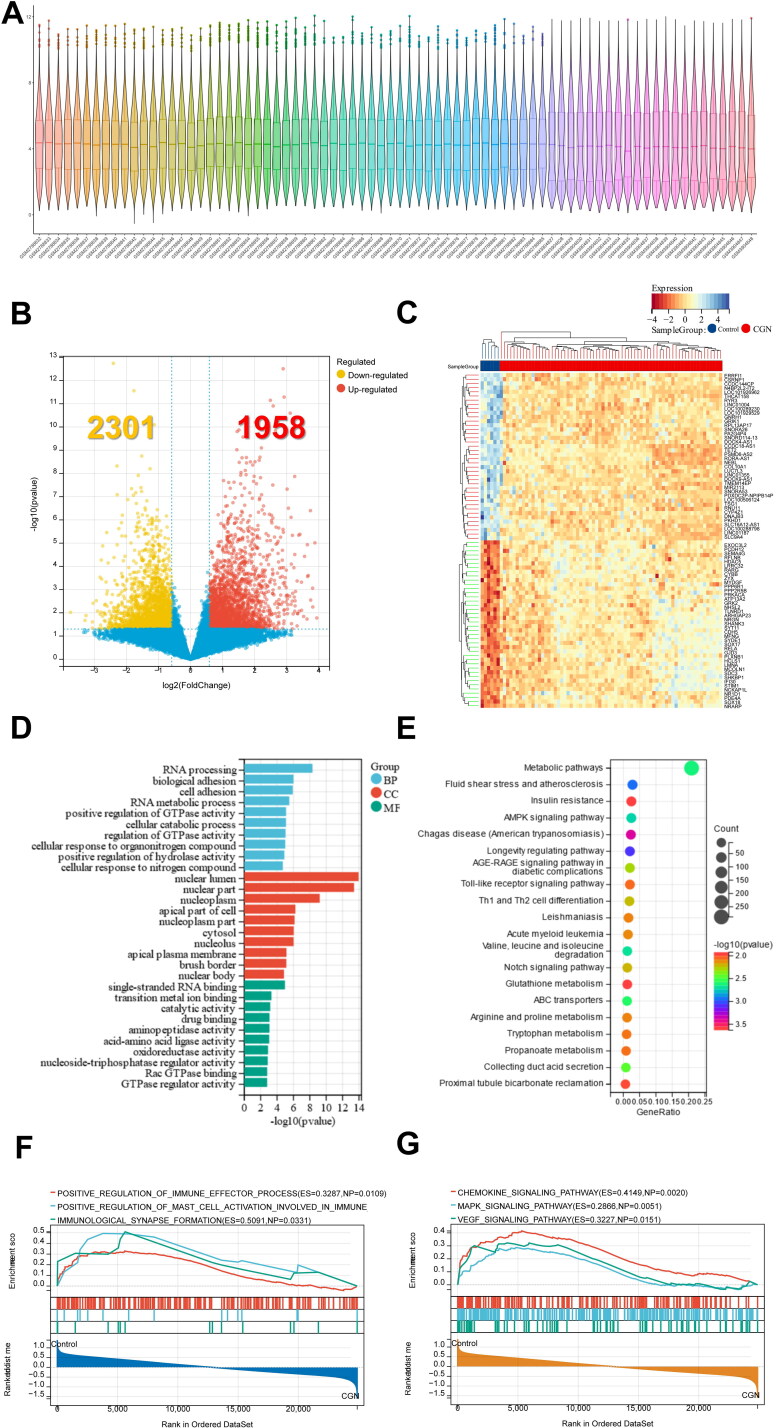
Identification and functional enrichment analysis of DE-mRNAs for CGN based on the GEO database. (A) Violin diagram shows the dataset sample distributions of GSE104066. (B,C) Volcano plot and cluster heat map show DE-mRNAs between the CGN and control group. (D) GO analysis for DE-mRNAs. (E) KEGG pathway analysis for DE-mRNAs. (F) GSEA GO biological processes analysis for DE-mRNAs. (G) GSEA KEGG pathway analysis for DE-mRNAs.

We obtained the GSE104066 expression matrix of CGN people from the GEO database with R software (Figure 4(A)) (Sup. File 6). A total of 4259 genes were screened as DE-mRNAs under the conditions of *p* value < 0.05 and | fold-change (FC) | > 1.5, with 1958 DE-mRNAs up-regulated and 2301 DE-mRNAs down-regulated (Figure 4(B,C)) (Sup. File 7). The biological functions and pathways related to 4259 DE-mRNAs were then examined using GO and KEGG analyses.

The top 10 GO BP results showed that RNA processing, biological adhesion, and metabolic processes of various enzymes, such as positive regulation of GTPase activity and positive regulation of hydrolase activity, were significantly enriched (Figure 4(D)). More importantly, the top 20 KEGG analyses showed that a large number of pathways related to immune and inflammatory responses were significantly enriched, including Th1 and Th2 cell differentiation, autophagy, Th17 cell differentiation, Notch signaling pathway, PPAR signaling pathway, and T cell receptor signaling pathway (Figure 4(E)).

In addition, the top 10 GSEA GO biological process results further revealed that DE-mRNAs regulated a variety of immunological responses, including positive regulation of immune effector processes, positive regulation of mast cell activation involved in immune RI, and immunological synapse (Figure 4(F)). Moreover, the top 10 GSEA KEGG results revealed that DE-mRNAs also regulated a large number of inflammatory responses, including chemokine signaling pathway, MAPK signaling pathway, and VEGF signaling pathway (Figure 4(G)). The findings above clearly imply that inflammation and autoimmunity are crucial components of CGN development.

### 3.4. Identification of clinically significant modules of CGN based on WGCNA

**Figure 5.**
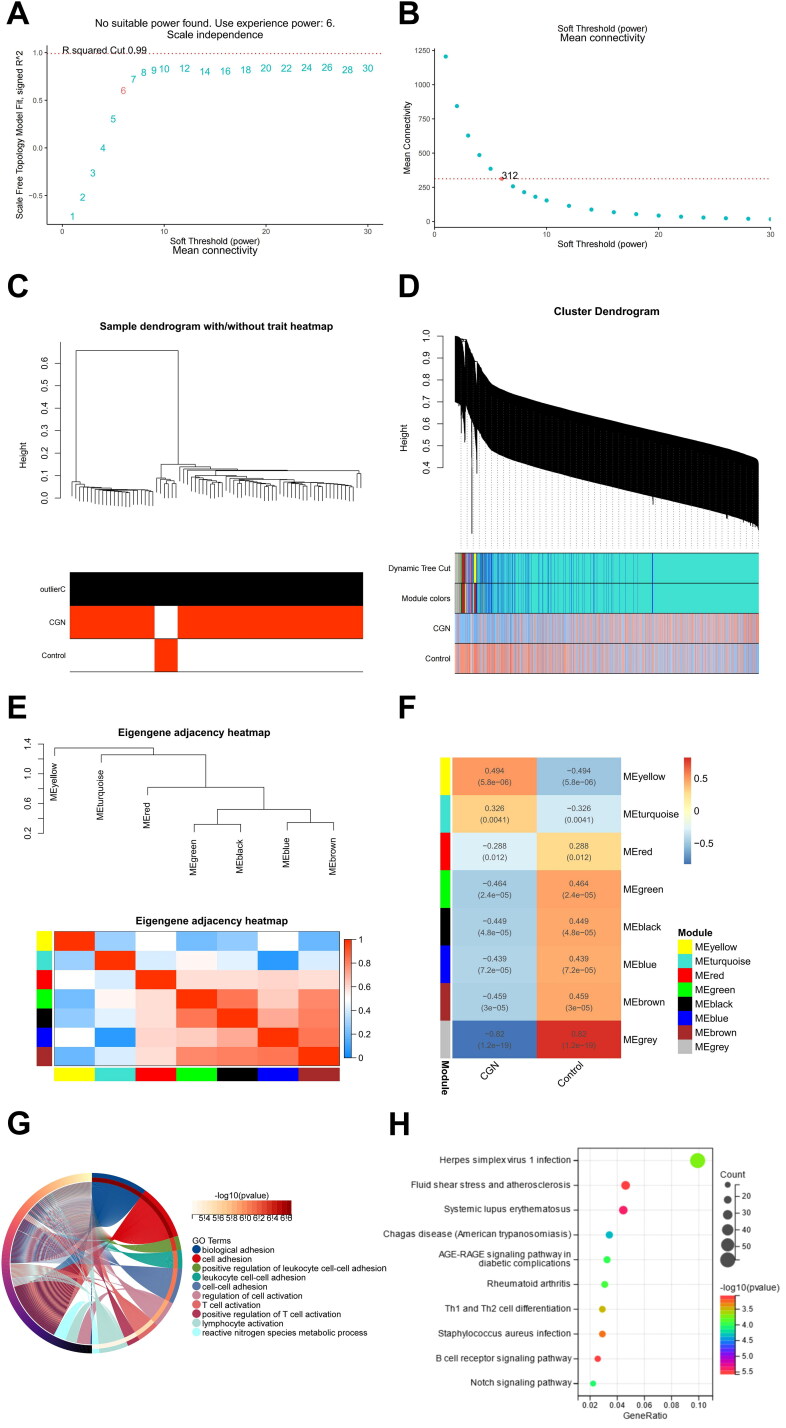
Identification of CGN-associated key modules Based on WGCNA analysis. (A,B) Scale-free fitting index analysis and mean connectivity of soft threshold power from 1 to 30. (C) Clustering dendrogram with tree leaves corresponding to individual samples. (D) Clustering dendrogram of all expressed genes based on a dissimilarity measure (1-TOM). (E) Correlation heatmap of module feature vector. (F) Correlation heatmap between module eigengene and CGN clinical trait. (G) GO biological processes analysis for turquoise module genes. (H) KEGG pathway analysis for turquoise module genes.

In order to determine the critical modules of DE-mRNAs most closely related to CGN, WGCNA was carried out using the GSE104066 gene expression profile (Sup. File 8). After combining strong association modules with a cluster height limit of 0.25 and excluding the obviously aberrant samples (Figure 5(A–C)), a total of 8 modules were found (Figure 5(D)). The clustering of module feature vectors was explored, and the results showed the distance between them (Figure 5(E)). Then, the relationships between the eight and clinical symptoms were also investigated. The results demonstrated the strongest correlation between the ‘group’ attribute (i.e. CGN and Control) and the yellow module, the turquoise module, the red module, the green module, the black module, the blue module, the brown module, and the grey module (Figure 5(F)).

To comprehend the biological roles that the eight modules genes perform, we carried out functional enrichment. According to the results of GO and KEGG analysis, DE-mRNAs in the turquoise module were related to numerous biological processes and pathways that were linked to infection, inflammation, and autoimmunity. GO enrichment analysis showed that the turquoise module’s genes were involved in positive regulation of leukocyte cell–cell adhesion, positive regulation of T cell activation, and lymphocyte activation (Figure 5(G)). More importantly, KEGG analysis was associated with herpes simplex virus 1 infection, Th1 and Th2 cell differentiation, B cell receptor signaling pathway, and Notch signaling pathway (Figure 5(H)), showing the potential pathogenesis of DE-mRNAs in the turquoise module in contributing to CGN.

### 3.5. Construction of the CircRNA-mediated CeRNA network

**Figure 6.**
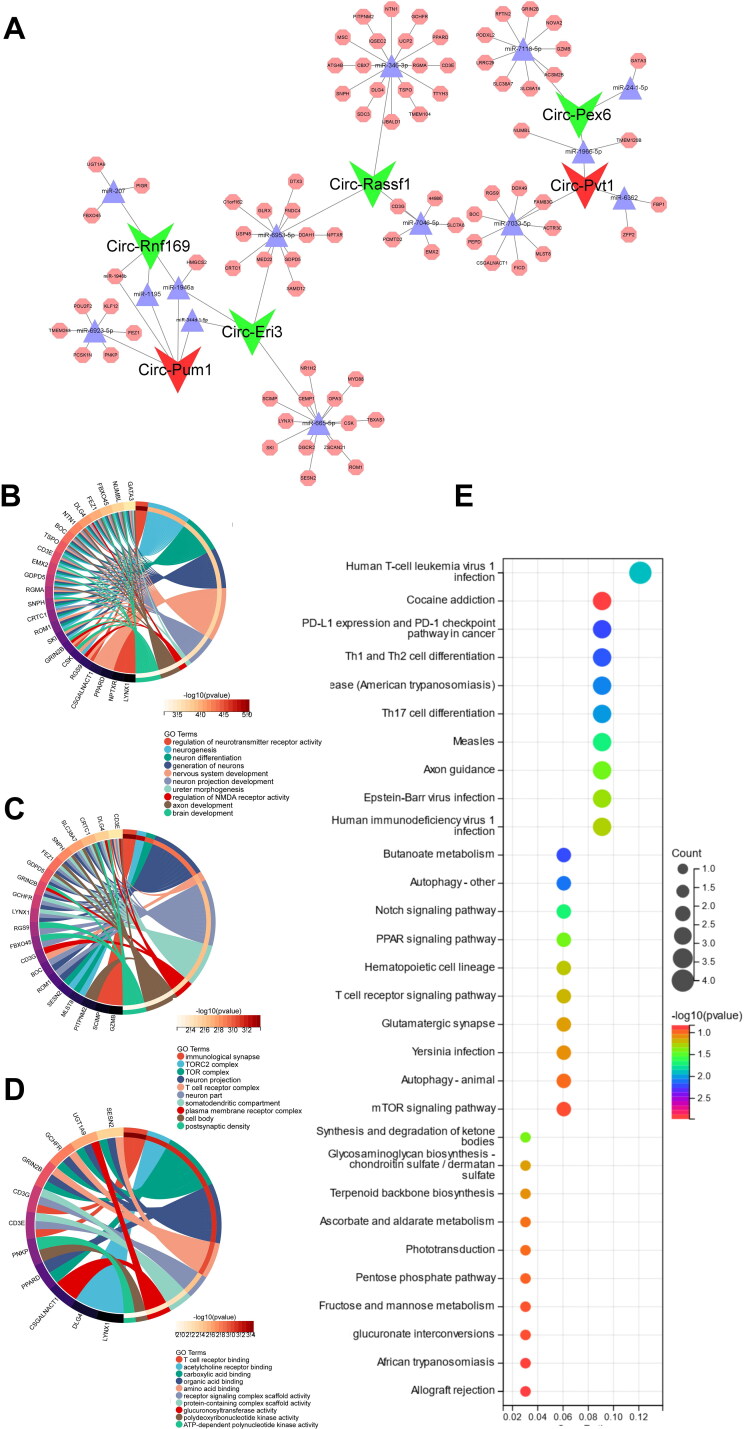
CeRNA regulatory network analysis Based on DE-circRNAs and DE-mRNAs. (A) The circRNA-mediated CeRNA regulation networks in CGN. (B) GO cellular components analysis for target mRNAs from the CeRNA network. (C) GO molecular functions analysis for target mRNAs from the CeRNA network. (D) GO biological processes analysis for target mRNAs from the CeRNA network. (E) KEGG pathway analysis for target mRNAs from the CeRNA network.

CircRNAs carry out a variety of functions in biological processes by interacting with different molecules, including RNA molecules. To explore the regulatory roles of circRNAs in mRNA expression *via* miRNA binding, we constructed a CeRNA regulatory network using 6 DE-CircRNAs detected by RNA-seq in MMCs, and 1747 DE-mRNAs identified *via* WGCNA turquoise module analysis in CGN patients from the GEO database. The network consisted of the top 5 miRNAs combined with screened circRNAs and mRNAs with high confidence (cumulative weighted context score cutoff level less than −0.5) bound to the miRNAs (Sup. Files 9–11), including 6 circRNAs, 38 miRNAs, and 80 mRNAs (Figure 6(A)).

To further investigate the potential role of these differentiated circular RNAs in CGN development, GO enrichment analysis was also performed to identify target mRNAs in the CeRNA network. The results showed that the top 10 cellular components are primarily involved in the immunological synapse, TOR complex, and T cell receptor complex (Figure 6(B)). In the molecular functions category, circRNAs were mainly related to T cell receptor binding, acetylcholine receptor binding, and carboxylic acid binding (Figure 6(C)). However, in the biological processes category, many terms related to regulation of neurotransmitter receptor activity, neurogenesis, neuron differentiation, and so on (Figure 6(D)).

More importantly, KEGG analysis indicated that a plethora of signaling pathways involved in CGN were significantly enriched, such as immune system-related Th1 and Th2 cell differentiation, Th17 cell differentiation, and T cell receptor signaling pathway; antiviral response-related human T-cell leukemia virus 1 infection, Epstein-Barr virus infection, and human immunodeficiency virus 1 infection; inflammation response-related Notch signaling pathway, PPAR signaling pathway, and mTOR signaling pathway (Figure 6(E)).

### 3.6. Identification of hub mRNAs from the CeRNA network based on the least absolute shrinkage and selection operator (LASSO)

**Figure 7.**
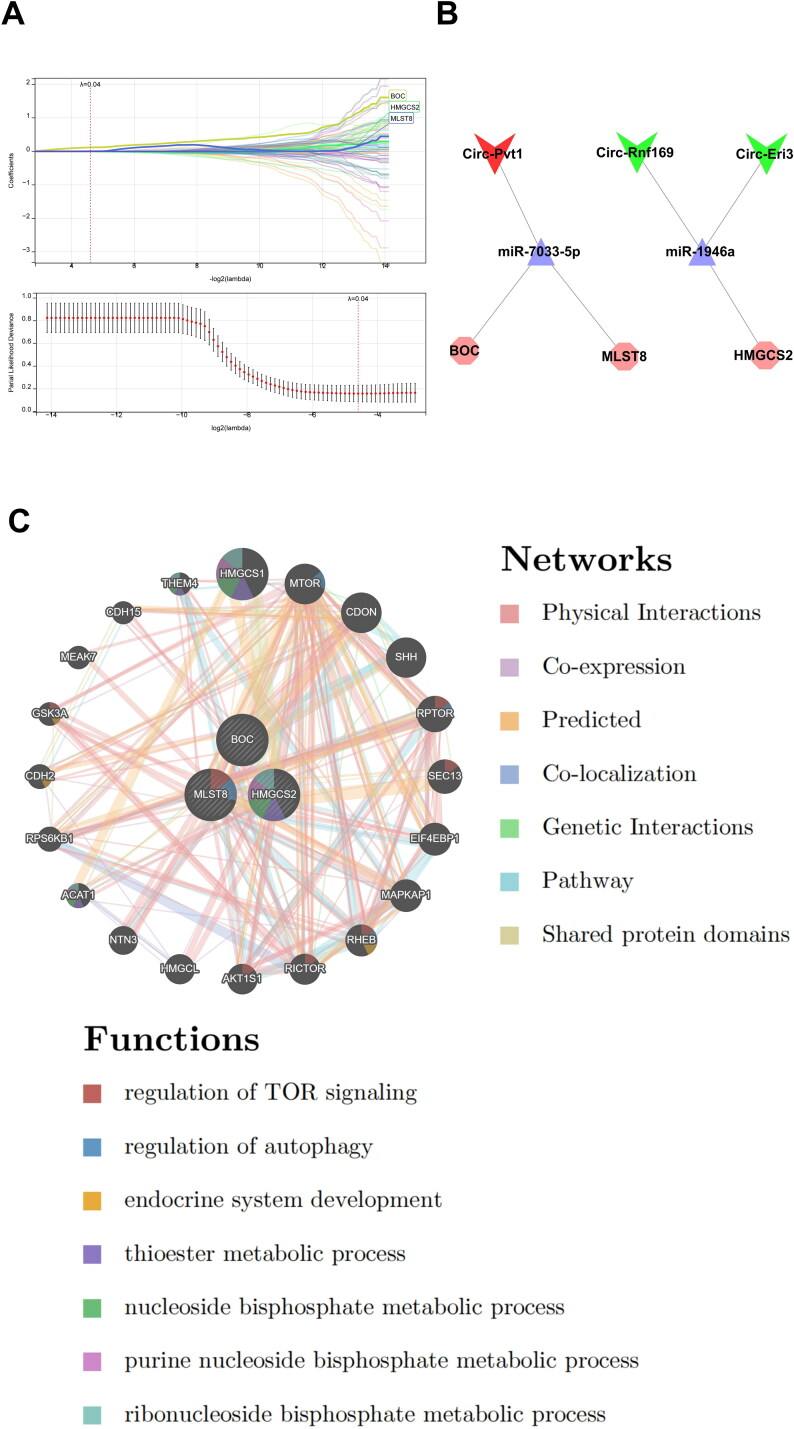
Identification of hub mRNAs from the CeRNA network. (A) Three feature genes with non-zero coefficients were selected by optimal lambda based on the LASSO regression model. (B) The hub CeRNA regulation networks in CGN. (C) GeneMANIA database showing functions and co-expression gene networks of hub mRNAs from the CeRNA network.

To further explore the CGN-associated hub ceRNA network and its relative mechanisms, we selected the candidate genes for feature gene screening through LASSO regression. The results of the LASSO regression identified three hub mRNAs (BOC, MLST8, and HMGCS2) with non-zero regression coefficients and the optimal lambda value of lambda. min = 0.04 (Figure 7(A)) (Sup. File 12). Finally, we identified 3 CircRNAs (Circ-Pvt1, Circ-Rnf169, and Circ-Eri3) and 2 miRNAs (miR-7033-5p and miR-1946a) that regulate the 3 hub mRNAs, and a hub CeRNA network was constructed (Figure 7(B)).

The GeneMANIA database was then used to study the hub mRNAs co-expression networks and probable roles (Figure 7(C)) (Sup. File 13). We discovered a complex PPI network with 0.60% protein domain, 1.88% pathway, 2.87% genetic relationships, 3.63% co-localization, 5.37% predicted interactions, 8.01% co-expression, and 77.64% physical interactions. According to a function study, they were mostly linked to regulation of TOR signaling, regulation of autophagy, endocrine system development, thioester metabolic process, nucleoside bisphosphate metabolic process, purine nucleoside bisphosphate metabolic process, and ribonucleoside bisphosphate metabolic process, indicating their critical involvement in the etiology of CGN.

### 3.7. Clinical correlations of hub mRNAs

**Figure 8.**
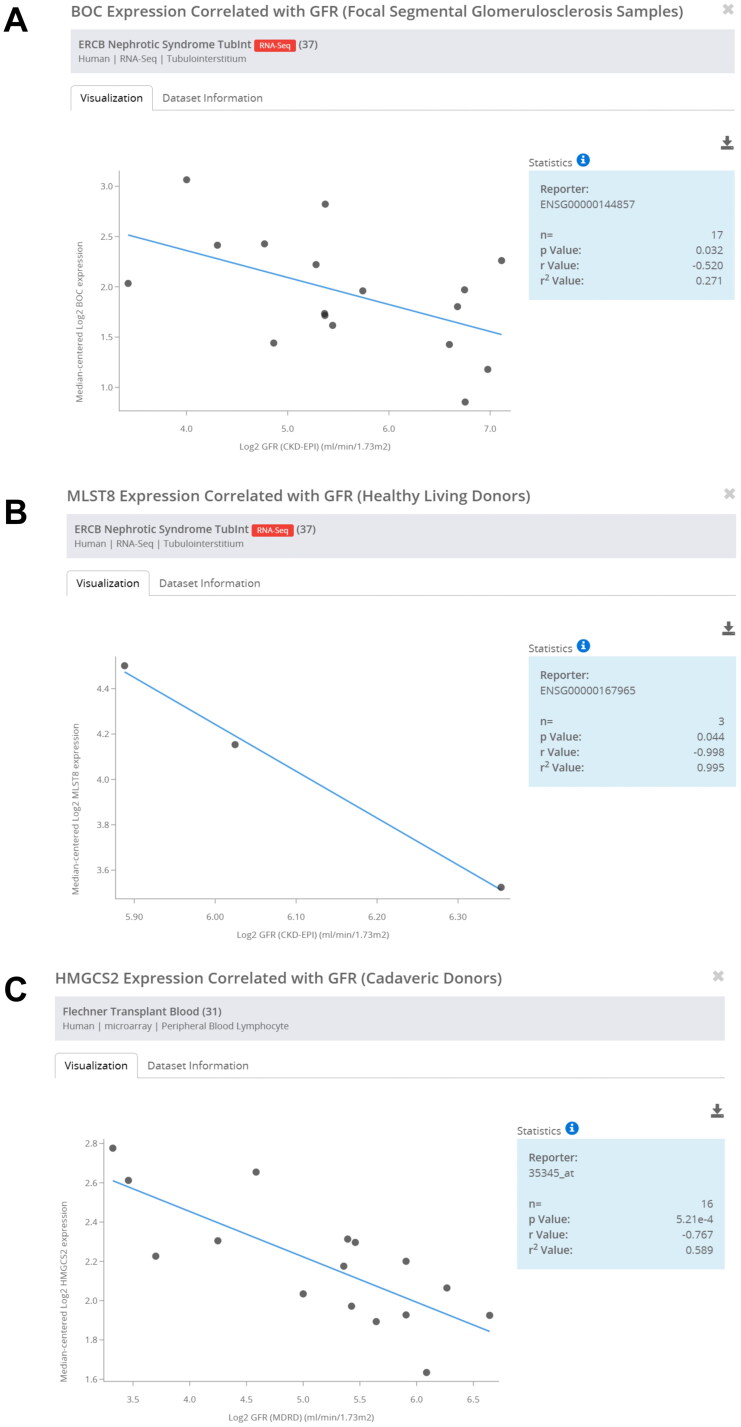
Correlation analysis of hub mRNAs and glomerular filtration rate. (A) Correlation analysis of BOC and glomerular filtration rate. (B) Correlation analysis of MLST8 and glomerular filtration rate. (C) Correlation analysis of HMGCS2 and glomerular filtration rate.

To explore the clinical implications of hub mRNAs in human CGN, we further analyzed the correlation between hub mRNAs expression and renal function. Data from the Nephroseq Database (https://www.nephroseq.org/) revealed that BOC, MLST8, HMGCS2 expression were all negatively correlated with the glomerular filtration rate (GFR) (mL/min.1.73 m^2^) in CGN patients (Figure 8(A–C)), demonstrating the important roles of BOC, MLST8, and HMGCS2 in the pathogenesis of CGN.

### 3.8. GSEA of hub mRNAs from the CeRNA network

**Figure 9.**
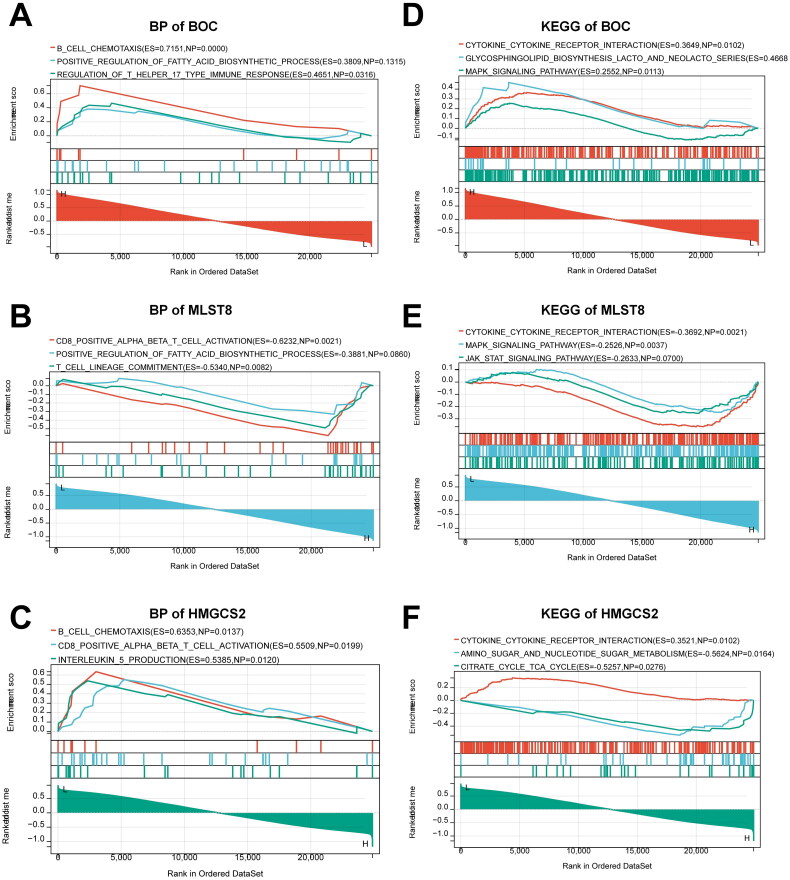
GSEA analysis of hub mRNAs from the CeRNA network. (A–C) GO biological processes analysis for (A) BOC, (B) MLST8, and (C) HMGCS2. (D-F) KEGG pathway analysis for (D) BOC, (E) MLST8, and (F) HMGCS2.

Furthermore, we investigated the precise signaling pathways and probable biological processes of the hub mRNAs from CeRNA network that regulated CGN development. The GSEA GO biological process results revealed that BOC regulated a variety of immunological responses, including B cell chemotaxis, regulation of T helper 17 type immune response, and immune response inhibiting signal transduction (Figure 9(A)). The main enriched terms for MLST8 were CD8 positive alpha beta T cell activation, B cell chemotaxis, and T cell lineage commitment (Figure 9(B)). The main enriched terms for HMGCS2 expression were CD8 positive alpha beta T cell activation, B cell chemotaxis, and IL-5 production (Figure 9(C)).

What’s more, the GSEA KEGG results revealed that BOC also regulated a large number of inflammatory responses, including cytokine-cytokine receptor interaction and MAPK signaling pathway (Figure 9(D)). The main enriched terms for MLST8 were cytokine-cytokine receptor interaction, MAPK signaling pathway, and JAK STAT signaling pathway (Figure 9(E)). As for HMGCS2, the main enriched terms were cytokine-cytokine receptor interaction, amino sugar and nucleotide sugar metabolism, and citrate cycle TCA cycle (Figure 9(F)). The above results suggest that all of these hub mRNAs from the CeRNA network might play essential roles in the regulation of immunity and inflammation in CGN.

### 3.9. Immune cell infiltration analysis

**Figure 10.**
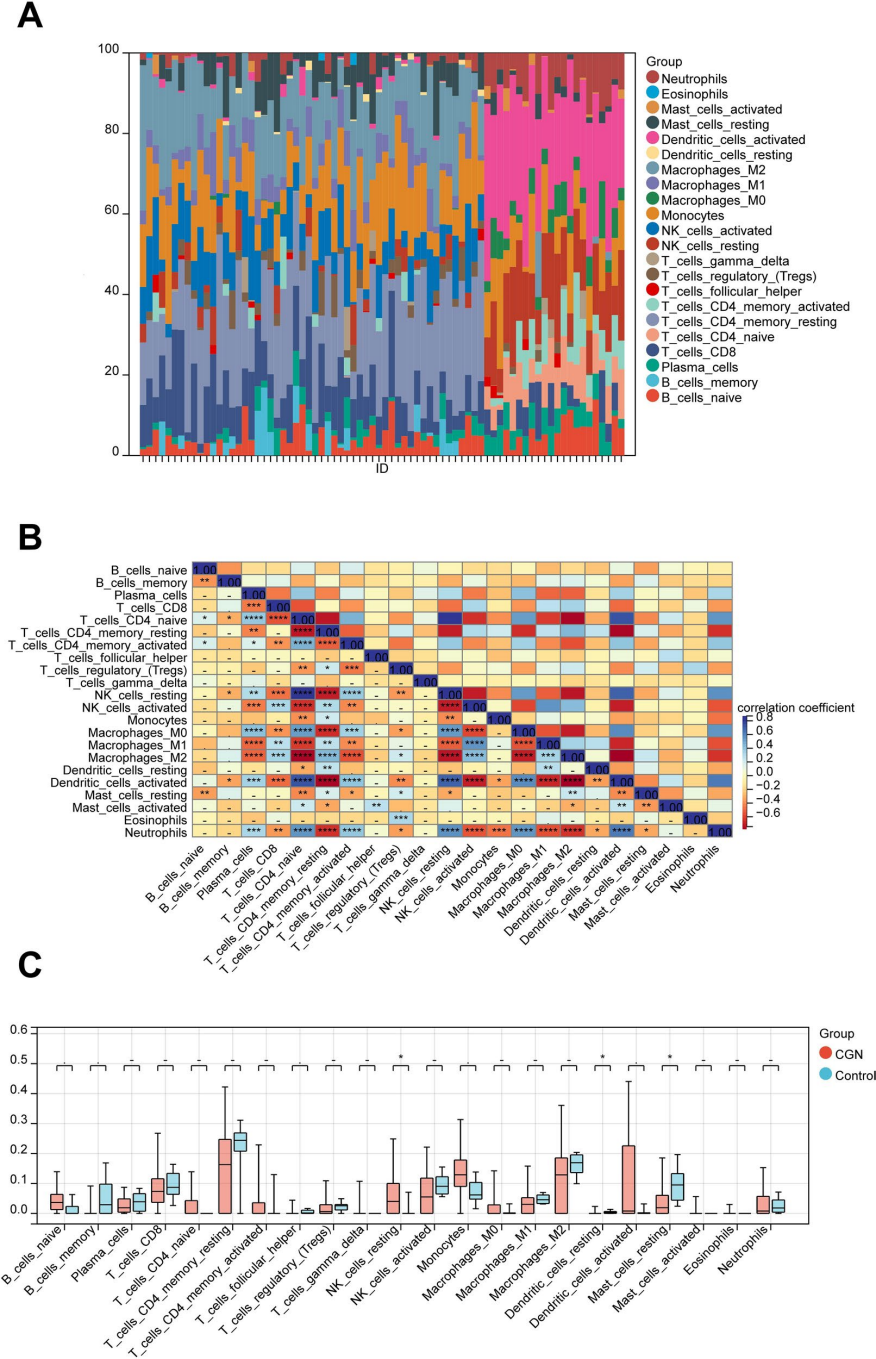
Immune infiltration analysis of CGN. (A) Each sample’s proportion of different immune cells. (B) Correlation between different immune cells (*p < 0.05, **p < 0.01). (C) Expression abundance of different immune cells in CGN and control (*p < 0.05, **p < 0.01).

To analyze immunological patterns in CGN and normal tissues, we used CIBERSORT to compute the proportion of 22 immune cells in each sample through the matrix of gene expression (Sup. File 14). Each sample’s 22 different categories of immune cells were represented by a histogram (Figure 10(A)). Each histogram’s colors showed the immune cell percentages, with a sum of 1 for each sample. The findings showed that the most prevalently infiltrated immune cells were monocytes (72), and the least infiltrated were eosinophils (4) in all 76 samples. In the subsequent study, the correlation between 22 categories of immuno-infiltrated cells in two groups was investigated (Figure 10(B)). The results showed a strong correlation between most of the immune cells. In addition, violin plots of the differential in immune cell infiltration revealed that, compared to the normal control sample, resting NK cells infiltrated more, whereas resting dendritic cells and resting mast cells infiltrated less (Figure 10(C)).

### 3.10. Correlation between hub mRNAs from the CeRNA network and immune cells

**Figure 11.**
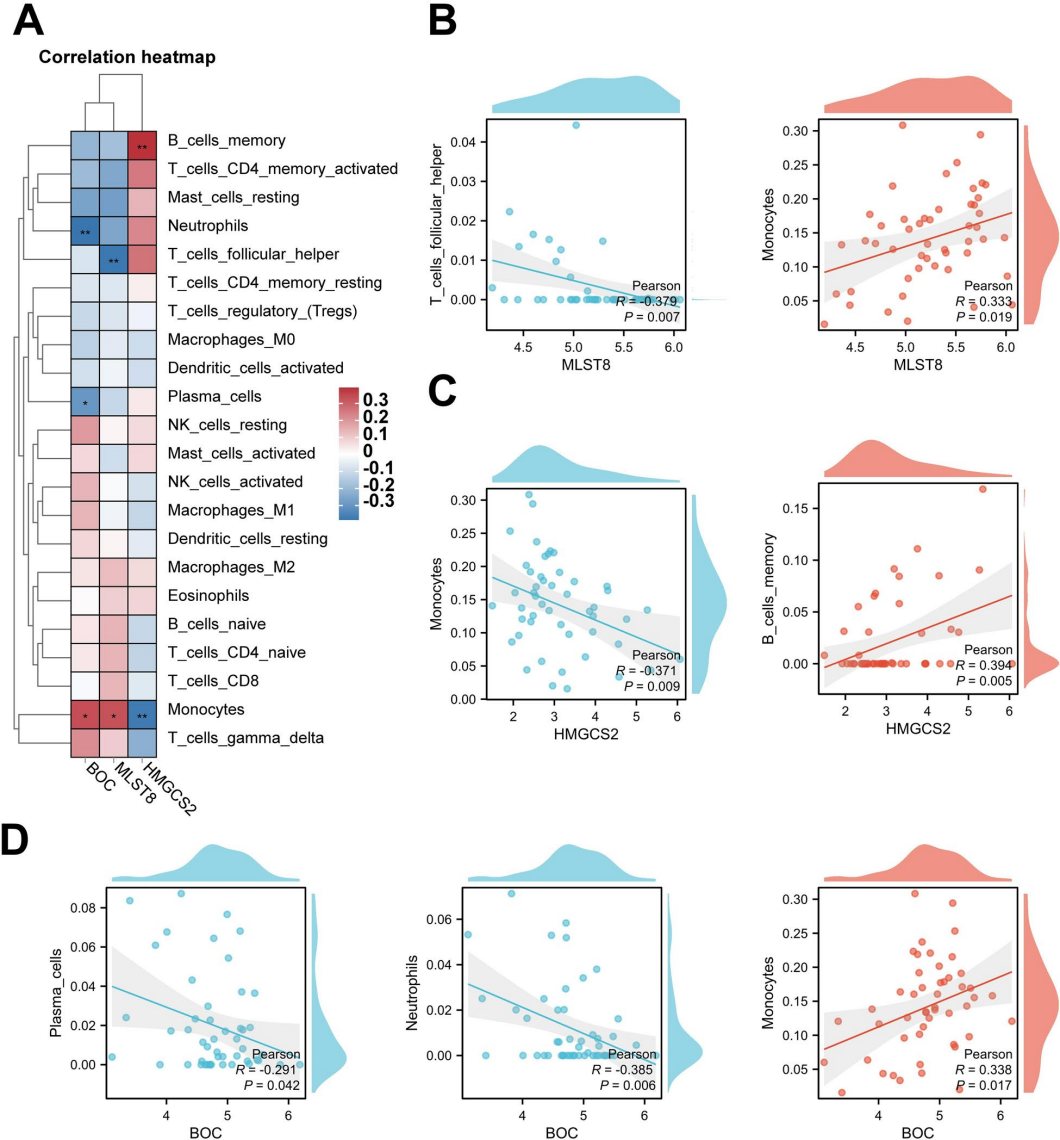
Correlation between the hub mRNAs from the CeRNA network and different immune cells. (A–D) Pearson’s correlation shows correlation between the hub mRNAs from the CeRNA network and different immune cells (*p < 0.05, **p < 0.01).

We next investigated the relationship between three hub mRNAs expression and immune cell abundance (Sup. File 15). The results of Pearson’s correlation showed hub mRNAs were associated with four types of immune cells (Figure 11(A)). As shown in Figure 11(B–D), BOC was statistically negatively correlated with neutrophils and plasma cells and positively correlated with monocytes; MLST8 was statistically negatively correlated with follicular helper T cells and positively correlated with monocytes; and HMGCS2 was statistically negatively correlated with monocytes and positively correlated with memory B cells, suggesting four types of immune cells may be regulated by hub mRNAs and play crucial roles in CGN formation.

## 4. Discussion

Transcripts with similar miRNA-binding sites, such as circRNAs, mRNAs, and lncRNAs, modulate cross-expression through competitive binding with miRNAs. These molecules form the CeRNA network, a precise and complex network of post-transcriptional regulation [14]. As a crucial component of the CeRNA networks, circRNA plays a crucial role in forming the circRNA-miRNA-mRNA axis, regulating various diseases, and being a common mechanism by which circRNA exerts its functions [15–17]. Mesangial cells play an essential role in renal autoimmunity and inflammatory disease processes, and mesangial cell-mediated glomerulonephritis is the main cause of ESRD [18,19]. To explore the potential contribution of circRNAs in the formation and development of CGN, we established an *in vitro* model of LPS-induced mesangial cell proliferation and inflammation, which is a widely utilized and standard CGN model [20,21]. Subsequently, MMC cells from LPS and control groups were analyzed by genome-wide RNA sequencing (RNA-seq) to identify the association between circRNAs and CGN. A total of 4868 circRNAs were detected in two groups of MMC cells, and they were observed to be enriched most in the exons (61.2%) of the genome region and on chromosome 5 of 22 chr. Further analysis identified 6 DE-circRNAs, of which 2 circRNAs were high expression and 4 circRNAs were low expression, revealing that these potential circRNAs may be involved in the CGN process.

To further investigate the possible functions of differential circRNAs in LPS-stimulated MMCs, we obtained a total of 1747 DE-mRNAs of the turquoise module from CGN people in the GEO database based on WGCNA analysis. Subsequently, we constructed a circRNAs-miRNAs-mRNAs CeRNA regulatory network, including 6 circRNAs, 38 miRNAs, and 80 mRNAs, using miRNA prediction software. GO and KEGG enrichment analysis revealed that target mRNAs from the CeRNA regulatory network were mainly related to the immune system, response to inflammation, and antiviral response, such as immune system-related Th1 and Th2 cell differentiation, Th17 cell differentiation, and T cell receptor signaling pathway; antiviral response-related human T-cell leukemia virus 1 infection, Epstein-Barr virus infection, and human immunodeficiency virus 1 infection; inflammation response-related Notch signaling pathway, PPAR signaling pathway, and mTOR ­signaling pathway.

To further identify the hub CeRNA network in LPS-stimulated MMCs, we selected target mRNAs from the CeRNA network using LASSO methods. Finally, 3 hub mRNAs (BOC, MLST8, and HMGCS2) were identified, and GSEA analysis revealed that they controlled a great deal of immune system responses and inflammatory pathways, including IL-5 production, MAPK signaling pathway, and JAK-STAT signaling pathway, indicating that BOC, MLST8, and HMGCS2 may play key roles in CGN development. IL-5 is responsible for regulating inflammation and immune suppression in the tumor microenvironment [22]. Its primary role is to promote the maturation of basophils and the activation of eosinophils [23]. Additionally, IL-5 has been shown to induce the formation of antigen-specific T-REG cells and prevent autoimmunity [24]. The MAPK signaling pathway plays a crucial role in kidney development, and inhibiting this pathway has been shown to improve kidney disease [25,26]. Specifically, the MAPK cascade promotes the proliferation of mesangial cells [27]. Mesangial cells are activated *via* the phosphorylation of three subfamilies of MAPKs – ERK1/2, JNK, and p38 MAPK – in response to high glucose stimulation [28]. The JAK/STAT signaling pathway, which includes the JAK and STAT gene families, is also implicated in many kidney diseases, such as diabetes and FSGS [29,30]. High glucose levels activate this pathway in mesangial cells, leading to mesangial cell proliferation and the accumulation of ECM) proteins [31]. Inhibiting JAK/STAT signaling in mesangial cells cultured in high glucose conditions can decrease the production of TGF-β1 and fibrin [32].

For a comprehensive understanding of the dysregulated immune cells in CGN, an immune infiltration investigation was conducted. We discovered that CGN tissue had higher levels of resting NK cells but lower levels of resting dendritic cells and resting mast cells. Moreover, our investigation demonstrated that hub mRNAs were statistically correlated with several main immune cells (neutrophils, plasma cells, monocytes, and follicular helper T cells). They might therefore be extremely important in the immunomodulation of CGN and be linked to the malfunctioning of inflammatory cells in CGN. Neutrophils, or polymorphonuclear neutrophils, are highly potent innate immune cells [33]. They play a significant role in the pathophysiology of kidney injury, whereby an activated neutrophil within the kidney can release products that may cause irreparable damage or even death [34]. Following ischemia-reperfusion, neutrophils are recruited to the kidney and become the primary effector cells of IRI [35]. During renal allograft infiltration, plasma cells – a type of B cell-derived cell – are found [36]. These plasma cells may begin in secondary lymphoid organs before aggregating and remaining in inflamed nonlymphoid organs, such as the kidney. This aggregation of plasma cells can increase the local concentration of antibodies and immune complexes, leading to enhanced kidney injury in SLE [37]. Studies have shown that plasma proteins accumulating in the mesangial region of the glomerulus can contribute to mesangial cell injury and proliferation, mesangial matrix production increase, and ultimately worsen glomerulosclerosis [38]. Monocytes, which originate from myeloid progenitors and differentiate throughout life, can progress from classical to intermediate to nonclassical monocytes [39,40]. Metabolic activation of monocytes is associated with oxidative stress, chronic inflammation, and kidney disease progression [41]. Following stimulation by inflammatory signals, circulating monocytes migrate and infiltrate the kidney, where they differentiate into macrophages [42]. Chemokines, such as MCP-1, released from mesangial cells, attract circulating monocytes to the kidney [43]. Mesangial cells are also expected to regulate the infiltration of neutrophils and monocytes into the kidney by upregulating adhesion molecules [44,45]. T follicular helper cells are a specific subset of T helper cells that are capable of producing IL-21 [46]. These cells, along with T helper 17 cells, are known to promote the production of autoantibodies and inflammation [47]. T follicular helper cells are also recognized as memory CD4 T helper cells that play a significant role in assisting B cells to generate high-affinity antibodies [48,49].

It should be admitted that there are still some limitations to our study. First, the number of samples tested in RNA-seq should be increased to reduce possible bias in sequencing results. Second, both circRNA-miRNA and miRNA-mRNA regulatory relationships were predicted by bioinformatics analysis, so all hypotheses and relevant mechanisms need to be verified by experiment. Third, the GEO dataset used in this study is derived from glomerular tissue, which may mask cell-type-specific expression changes. Further studies are warranted to overcome the limitations mentioned above.

## 5. Conclusions

Our research provided a transcriptome-wide map of mesangial cell-derived circRNAs, revealing a potential relationship between circRNAs and CGN. Besides, the circRNA-miRNA-mRNA network and their potential biological functions in CGN were successfully constructed and systematically analyzed. Overall, the results of our study have unveiled a novel mechanism of circRNA regulation in mesangial cell proliferation and inflammation, which has important implications for our understanding of the pathogenesis of CGN.

## Supplementary Material

Supplemental Material

## Data Availability

The data used and analyzed to support the findings of this study are available from the corresponding author upon request.

## References

[1] Floege J, Amann K. Primary glomerulonephritides. Lancet. 2016;387(10032):1–19. doi: 10.1016/S0140-6736(16)00272-5. 26921911

[2] Zhang L, Long J, Jiang W, et al. Trends in chronic kidney disease in China. N Engl J Med. 2016;375(9):905–906. doi: 10.1056/NEJMc1602469. 27579659

[3] Sethi S, Fervenza FC. Standardized classification and reporting of glomerulonephritis. Nephrol Dial Transplant. 2019;34(2):193–199. doi: 10.1093/ndt/gfy220. 30124958

[4] Yu H, Artomov M, Brähler S, et al. A role for genetic susceptibility in sporadic focal segmental glomerulosclerosis. J Clin Invest. 2016;126(3):1067–1078. doi: 10.1172/JCI82592. 26901816 PMC4767358

[5] Shimizu A, Kitamura H, Masuda Y, et al. Apoptosis in the repair process of experimental proliferative glomerulonephritis. Kidney Int. 1995;47(1):114–121. doi: 10.1038/ki.1995.13. 7731136

[6] Baker AJ, Mooney A, Hughes J, et al. Mesangial cell apoptosis: the major mechanism for resolution of glomerular hypercellularity in experimental mesangial proliferative nephritis. J Clin Invest. 1994;94(5):2105–2116. doi: 10.1172/JCI117565. 7962557 PMC294654

[7] Zhong Y, Du Y, Yang X, et al. Circular RNAs function as ceRNAs to regulate and control human cancer progression. Mol Cancer. 2018;17(1):79. doi: 10.1186/s12943-018-0827-8. 29626935 PMC5889847

[8] Guo JU, Agarwal V, Guo H, et al. Expanded identification and characterization of mammalian circular RNAs. Genome Biol. 2014;15(7):409. doi: 10.1186/PREACCEPT-1176565312639289. 25070500 PMC4165365

[9] Han D, Li J, Wang H, et al. Circular RNA circMTO1 acts as the sponge of microRNA-9 to suppress hepatocellular carcinoma progression. Hepatology. 2017;66(4):1151–1164. doi: 10.1002/hep.29270. 28520103

[10] Ritchie ME, Phipson B, Wu D, et al. limma powers differential expression analyses for RNA-sequencing and microarray studies. Nucleic Acids Res. 2015;43(7):e47–e47. doi: 10.1093/nar/gkv007. 25605792 PMC4402510

[11] Zhang X, Chen X, Wu D, et al. Downregulation of connexin 43 expression by high glucose induces senescence in glomerular mesangial cells. J Am Soc Nephrol. 2006;17(6):1532–1542. doi: 10.1681/ASN.2005070776. 16675599

[12] Subramanian A, Tamayo P, Mootha VK, et al. Gene set enrichment analysis: a knowledge-based approach for interpreting genome-wide expression profiles. Proc Natl Acad Sci USA. 2005;102(43):15545–15550. doi: 10.1073/pnas.0506580102. 16199517 PMC1239896

[13] Chen B, Khodadoust MS, Liu CL, et al. Profiling Tumor Infiltrating Immune Cells with CIBERSORT. Methods Mol Biol. 2018;1711:243–259. 29344893 10.1007/978-1-4939-7493-1_12 PMC5895181

[14] Tay Y, Rinn J, Pandolfi PP. The multilayered complexity of ceRNA crosstalk and competition. Nature. 2014;505(7483):344–352. doi: 10.1038/nature12986. 24429633 PMC4113481

[15] Bi W, Huang J, Nie C, et al. CircRNA circRNA_102171 promotes papillary thyroid cancer progression through modulating CTNNBIP1-dependent activation of β-catenin pathway. J Exp Clin Cancer Res. 2018;37(1):275. doi: 10.1186/s13046-018-0936-7. 30424816 PMC6234664

[16] Li H, Xu JD, Fang XH, et al. Circular RNA circRNA_000203 aggravates cardiac hypertrophy via suppressing miR-26b-5p and miR-140-3p binding to Gata4. Cardiovasc Res. 2020;116(7):1323–1334. doi: 10.1093/cvr/cvz215. 31397837 PMC7243276

[17] Sang Y, Chen B, Song X, et al. circRNA_0025202 Regulates tamoxifen sensitivity and tumor progression via regulating the miR-182-5p/FOXO3a axis in breast cancer. Mol Ther. 2019;27(9):1638–1652. doi: 10.1016/j.ymthe.2019.05.011. 31153828 PMC6731174

[18] Yu H, Cui S, Mei Y, et al. Mesangial cells exhibit features of antigen-presenting cells and activate CD4+ T cell responses. J Immunol Res. 2019;2019:1–14. doi: 10.1155/2019/2121849. PMC6604415 31317046

[19] Guo L, Luo S, Du Z, et al. Targeted delivery of celastrol to mesangial cells is effective against mesangioproliferative glomerulonephritis. Nat Commun. 2017;8(1):878. doi: 10.1038/s41467-017-00834-8. 29026082 PMC5638829

[20] Shushakova N, Tkachuk N, Dangers M, et al. Urokinase-induced activation of the gp130/Tyk2/Stat3 pathway mediates a pro-inflammatory effect in human mesangial cells via expression of the anaphylatoxin C5a receptor. J Cell Sci. 2005;118(12):2743–2753. doi: 10.1242/jcs.02409. 15944400

[21] Gao J, Wei L, Song J, et al. In vitro and in vivo study of the expression of the Syk/Ras/c‑Fos pathway in chronic glomerulonephritis. Mol Med Rep. 2018;18(4):3683–3690. 30106104 10.3892/mmr.2018.9355 PMC6131599

[22] Dougan M, Dranoff G, Dougan SK. GM-CSF, IL-3, and IL-5 family of cytokines: regulators of inflammation. Immunity. 2019;50(4):796–811. doi: 10.1016/j.immuni.2019.03.022. 30995500 PMC12512237

[23] Mohammadpour R, Yazdimamaghani M, Cheney DL, et al. Subchronic toxicity of silica nanoparticles as a function of size and porosity. J Control Release. 2019;304:216–232. doi: 10.1016/j.jconrel.2019.04.041. 31047961 PMC6681828

[24] Gao P, Uzun Y, He B, et al. Risk variants disrupting enhancers of TH1 and TREG cells in type 1 diabetes. Proc Natl Acad Sci USA. 2019;116(15):7581–7590. doi: 10.1073/pnas.1815336116. 30910956 PMC6462079

[25] Ihermann-Hella A, Hirashima T, Kupari J, et al. Dynamic MAPK/ERK activity sustains nephron progenitors through niche regulation and primes precursors for differentiation. Stem Cell Rep. 2018;11(4):912–928. doi: 10.1016/j.stemcr.2018.08.012. PMC6178244 30220628

[26] Pengal R, Guess AJ, Agrawal S, et al. Inhibition of the protein kinase MK-2 protects podocytes from nephrotic syndrome-related injury. Am J Physiol Renal Physiol. 2011;301(3):F509–F519. doi: 10.1152/ajprenal.00661.2010. 21613416 PMC3174552

[27] Kim D, Li HY, Lee JH, et al. Lysophosphatidic acid increases mesangial cell proliferation in models of diabetic nephropathy via Rac1/MAPK/KLF5 signaling. Exp Mol Med. 2019;51(2):1–10. doi: 10.1038/s12276-019-0217-3. PMC6377648 30770784

[28] Fang S, Jin Y, Zheng H, et al. High glucose condition upregulated Txnip expression level in rat mesangial cells through ROS/MEK/MAPK pathway. Mol Cell Biochem. 2011;347(1–2):175–182. doi: 10.1007/s11010-010-0626-z. 20953987

[29] Wang X, Liao X, Yu T, et al. Analysis of clinical significance and prospective molecular mechanism of main elements of the JAK/STAT pathway in hepatocellular carcinoma. Int J Oncol. 2019;55(4):805–822. doi: 10.3892/ijo.2019.4862. 31485610 PMC6741847

[30] Tao J, Mariani L, Eddy S, et al. JAK-STAT activity in peripheral blood cells and kidney tissue in IgA nephropathy. Clin J Am Soc Nephrol.. 2020;15(7):973–982. doi: 10.2215/CJN.11010919. 32354727 PMC7341773

[31] Marrero MB, Banes-Berceli AK, Stern DM, et al. Role of the JAK/STAT signaling pathway in diabetic nephropathy. Am J Physiol Renal Physiol. 2006;290(4):F762–F768. doi: 10.1152/ajprenal.00181.2005. 16527921

[32] Boengler K, Hilfiker-Kleiner D, Drexler H, et al. The myocardial JAK/STAT pathway: from protection to failure. Pharmacol Ther. 2008;120(2):172–185. doi: 10.1016/j.pharmthera.2008.08.002. 18786563

[33] Jin Y, Dixon B, Jones L, et al. The differential reactive oxygen species production of tear neutrophils in response to various stimuli in vitro. Int J Mol Sci.. 2021;22(23):12899. doi: 10.3390/ijms222312899. 34884704 PMC8657846

[34] Jaeschke H. Mechanisms of Liver Injury. II. Mechanisms of neutrophil-induced liver cell injury during hepatic ischemia-reperfusion and other acute inflammatory conditions. Am J Physiol Gastrointest Liver Physiol. 2006;290(6):G1083–G1088. doi: 10.1152/ajpgi.00568.2005. 16687579

[35] Ferhat M, Robin A, Giraud S, et al. Endogenous IL-33 contributes to kidney ischemia-reperfusion injury as an alarmin. J Am Soc Nephrol.. 2018;29(4):1272–1288. doi: 10.1681/ASN.2017060650. 29436517 PMC5875946

[36] Hasegawa J, Honda K, Omoto K, et al. Clinical and pathological features of plasma cell-rich acute rejection after kidney transplantation. Transplantation. 2018;102(5):853–859. doi: 10.1097/TP.0000000000002041. 29319615

[37] Cheng D, Luo Z, Fu X, et al. Elevated cerebrospinal fluid anti-CD4 autoantibody levels in HIV associate with neuroinflammation. Microbiol Spectr. 2022;10(1):e0197521. doi: 10.1128/spectrum.01975-21. 34985329 PMC8729763

[38] Burton C, Harris KP. The role of proteinuria in the progression of chronic renal failure. Am J Kidney Dis. 1996;27(6):765–775. doi: 10.1016/S0272-6386(96)90512-0. 8651239

[39] Rogacev KS, Zawada AM, Hundsdorfer J, et al. Immunosuppression and monocyte subsets. Nephrol Dial Transplant. 2015;30(1):143–153. doi: 10.1093/ndt/gfu315. 25313167

[40] Yona S, Kim KW, Wolf Y, et al. Fate mapping reveals origins and dynamics of monocytes and tissue macrophages under homeostasis [published correction appears in Immunity. Immunity. 2013;38(1):79–91. doi: 10.1016/j.immuni.2012.12.001. 23273845 PMC3908543

[41] Zeng H, Wang L, Li J, et al. Single-cell RNA-sequencing reveals distinct immune cell subsets and signaling pathways in IgA nephropathy. Cell Biosci. 2021;11(1):203. doi: 10.1186/s13578-021-00706-1. 34895340 PMC8665497

[42] Zhou D, Huang C, Lin Z, et al. Macrophage polarization and function with emphasis on the evolving roles of coordinated regulation of cellular signaling pathways. Cell Signal. 2014;26(2):192–197. doi: 10.1016/j.cellsig.2013.11.004. 24219909

[43] Menzies RI, Booth JWR, Mullins JJ, et al. Hyperglycemia-induced renal P2X7 receptor activation enhances diabetes-related injury. EBioMedicine. 2017;19:73–83. doi: 10.1016/j.ebiom.2017.04.011. 28434946 PMC5440600

[44] Wuthrich RP. Intercellular adhesion molecules and vascular cell adhesion molecule-1 and the kidney. J Am Soc Nephrol. 1992;3(6):1201–1211. doi: 10.1681/ASN.V361201. 1282378

[45] Menè P, Fais S, Cinotti GA, et al. Regulation of U-937 monocyte adhesion to cultured human mesangial cells by cytokines and vasoactive agents. Nephrol Dial Transplant. 1995;10(4):481–489. doi: 10.1093/ndt/10.4.481. 7542754

[46] Rayasam A, Kijak JA, Kissel L, et al. CXCL13 expressed on inflamed cerebral blood vessels recruit IL-21 producing TFH cells to damage neurons following stroke. J Neuroinflammation. 2022;19(1):125. doi: 10.1186/s12974-022-02490-2. 35624463 PMC9145182

[47] Naskar D, Teng F, Felix KM, et al. Synthetic retinoid AM80 ameliorates lung and arthritic autoimmune responses by inhibiting T follicular helper and Th17 cell responses. J Immunol. 2017;198(5):1855–1864. doi: 10.4049/jimmunol.1601776. 28130500 PMC5324833

[48] Crotty S. Follicular helper CD4 T cells (TFH). Annu Rev Immunol. 2011;29(1):621–663. doi: 10.1146/annurev-immunol-031210-101400. 21314428

[49] Fazilleau N, Mark L, McHeyzer-Williams LJ, et al. Follicular helper T cells: lineage and location. Immunity. 2009;30(3):324–335. doi: 10.1016/j.immuni.2009.03.003. 19303387 PMC2731675

