# Supplementary figures and images for: Mesangial cell-derived CircRNAs in chronic glomerulonephritis: RNA sequencing and bioinformatics analysis
Source: Ren Fail. 2024 Jul 1;46(2):2371059. doi: 10.1080/0886022X.2024.2371059 (PMC467094; doi:10.1080/0886022X.2024.2371059)

# Flow Cytometry

## Control 1

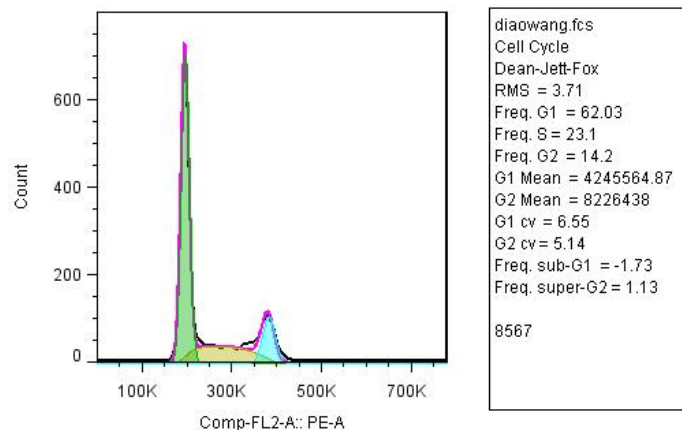

## Control 2

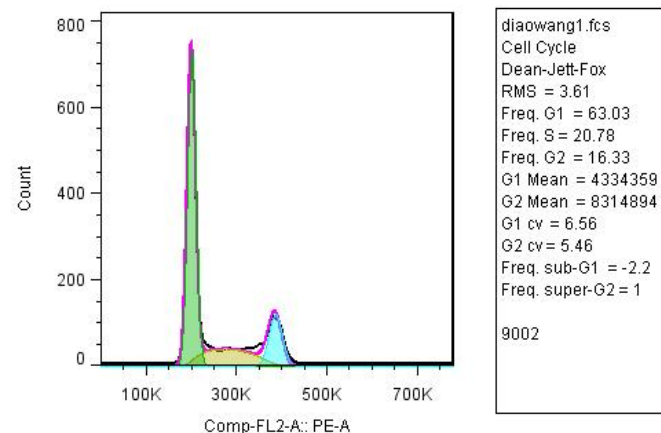

## Control 3

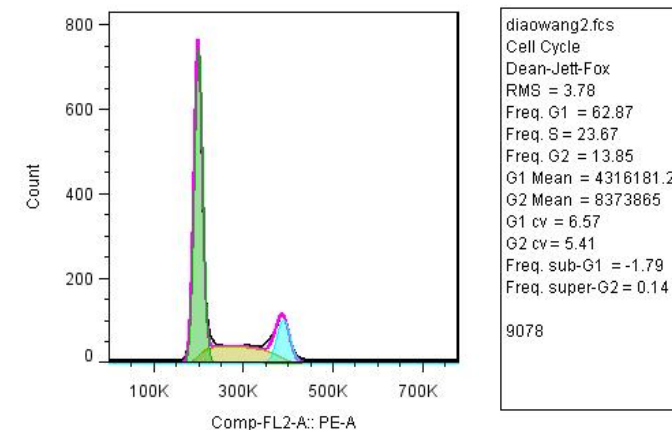

## LPS 1

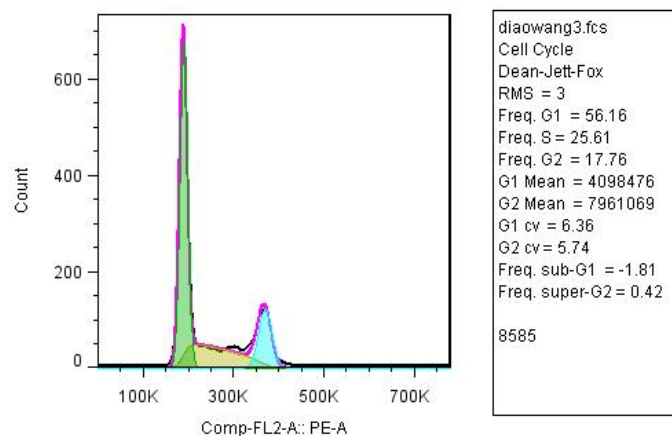

## LPS 2

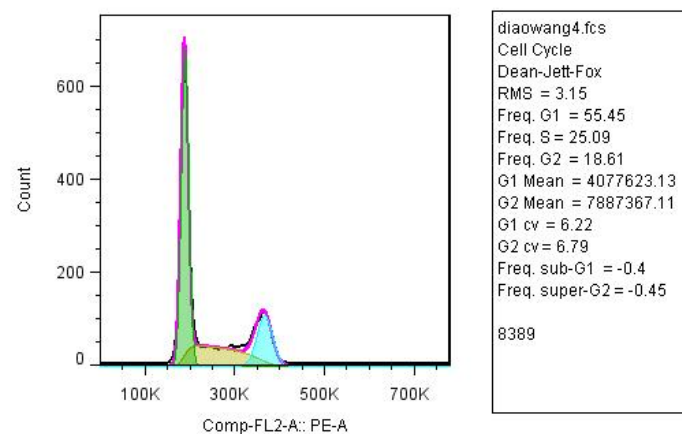

## LPS 3

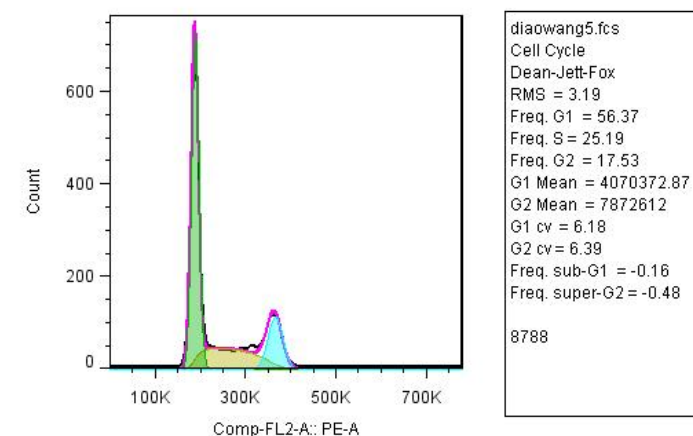

Supplement: Supplemental Material [file IRNF_A_2371059_SM1376.zip › Supplementary File 2.pdf]
